# Social support and country-level factors associated with cancer screening participation in 18 European countries: a multilevel analysis of the European Health Interview Survey

**DOI:** 10.1186/s13690-026-02023-w

**Published:** 2026-08-06

**Authors:** Maelodee Chong Armstrong, Mattias Johansson, Megumi Rosenberg, Nawi Ng

**Affiliations:** 1 School of Public Health and Community Medicine, Institution of Medicine, Sahlgrenska Academy, University of Gothenburg, Gothenburg, Sweden; 2 Early detection, Prevention, and Infections Branch (EPR), International Agency for Research on Cancer (IARC/WHO), Lyon, France; 3 World Health Organization (WHO), 20 Avenue Appia, Geneva, 1211 Switzerland

**Keywords:** Cancer screening, Colorectal cancer, Breast cancer, Cervical cancer, Social support, Europe, Multilevel model

## Abstract

**Background:**

Amid the rising burden of cancer in Europe, participation in colorectal, breast, and cervical cancer screening programmes remains inadequate. The potential role of social support in increasing screening uptake across different types of cancer screening and within multinational contexts remains underexplored.

**Methods:**

Two-level multilevel analyses were conducted using data from the European Health Interview Survey Wave 3 in 18 European countries. We analysed data from subsamples of participants who responded to the questions for colorectal (*n* = 65,089), breast (*n* = 35,281), and cervical (*n* = 36,763) cancer screening. Social support was measured using the Oslo-3 Social Support Scale. Participants were classified as overdue for screening according to cancer-specific guidelines for different target age groups.

**Results:**

The overall prevalence of being overdue was 43% for colorectal, 31% for breast, and 19% for cervical tests; however, substantial variation was observed between countries (i.e., from 22% in Denmark to 72% in Greece for colorectal, 4% in Sweden to 57% in Estonia for breast, and 6% in Sweden to 43% in the Netherlands for cervical screenings). Stronger social support was associated with a greater likelihood of being up to date with cancer screening, with stronger associations observed for breast and cervical cancers than for colorectal cancer. Country-level factors, including screening programme availability, social progress, out-of-pocket payments, and general practitioner density, explained nearly half of the variation in breast and colorectal cancer screening uptake, but less so for cervical cancer.

**Conclusions:**

Interventions promoting screening uptake should consider strengthening individual-level social support and addressing contextual-level structural barriers.

**Supplementary Information:**

The online version contains supplementary material available at 10.1186/s13690-026-02023-w.

**Table:** 

Text box 1. Contributions to the Literature
• This study quantifies the suboptimal and unequal screening uptake for breast, cervical, and colorectal cancer across 18 European countries using the latest available data from the European Health Interview Survey.
• Demonstrates how individuals with poor social support were more likely to be overdue for cancer screenings and extends prior research by comparing the strength of this association across cancer types.
• Highlights the influence of country-level factors related to screening uptake and underscores the critical need for countries to address structural barriers to colorectal screening participation by implementing organised, population-based programmes.

## Background

Cancer accounts for nearly one in six deaths worldwide - a mortality rate that could be greatly reduced with early detection and treatment [1]. Organised screening enables the detection of cancer at earlier stages, when less aggressive treatment is more effective [2–3]. However, prevention service policies, provision, and uptake differ substantially between European countries. The International Agency for Research on Cancer (IARC) [2] reported in 2017 that population-based screening programmes were not fully established in all member states and that average participation rates across the European Union (EU) for breast (60%), cervical (51%), and colorectal (38%) cancer screening remained below the respective acceptable standards of 70%, 70% and 45%.

Lagging participation rates result from diverse and interrelated factors from individual and healthcare system levels [4]. The Behavioural Model of Health Services Use [5] illustrates how enabling resources such as social support could affect individuals’ health behaviours. Studies have identified positive associations between the social support and uptake of breast [6–7], cervical [7], and colorectal [6] cancer screening. Yet other predisposing characteristics may influence both individuals’ level of social support and perception of health status, indirectly affecting healthcare utilisation [5]. These are individual level characteristics such as lower education levels [8–9], being unemployed [4], younger ages and those residing in rural areas [10], worse self-rated health [8], living with comorbidities [11], and worse mental health [12] which have been found to be associated with lower cancer screening uptake. Particularly in the case of colorectal screening, males are less likely to attend than females [13–14]. Furthermore, certain household types, for example being without a partner [10] or living alone [14], are associated with lower cancer screening uptake. The magnitude and direction of these associations tend to differ across cancer types [15], suggesting the potential of comparing analyses of social support across cancers.

Moreover, recent work has underscored the importance of examining higher-level contextual factors [14]. Country-level factors, such as the availability of healthcare resources and organisation systems, further influences health behaviour [5]. In countries without or with recently implemented screening programmes, commonly cited barriers to participation include limited information and service unavailability, whereas in countries with long-established national programmes with high screening rates, non-participation was solely linked to the belief that screening was unnecessary [16]. Greater healthcare accessibility—measured by indicators such as lower household out-of-pocket expenditures and higher general practitioner (GP) density—has been shown to reduce income- and education-related disparities in screening uptake [17–18]. The relationship between non-economic dimensions of societal wellbeing and screening uptake is underexplored. Social Progress Index (SPI), an example of the non-economic dimensions of societal wellbeing, provides a comprehensive cross-country measure of basic human needs, foundations of wellbeing (e.g., education and health), and opportunities (e.g., equality of rights) [19]. The multilevel conceptual framework of factors associated with screening programme uptake is shown in Supplementary Fig. 1[F1].

The role of social support in cancer screening is vital yet often overlooked, as research has largely focused on socioeconomic and demographic factors. While inequalities in screening participation of cervical [9], breast [20], and colorectal [10, 14] cancer have been recently studied in multinational European studies, only one investigated the role of social support [14]. The previous study [14], which used Wave 2 of the European Health Interview Survey (EHIS), did not account for the hierarchical structure of the data and the potential influence of contextual country-level factors related to screening programme uptake. The present study allows for a more up-to-date and comprehensive assessment of the social and contextual determinants of cancer screening participation across Europe by assessing the three types of cancer screening within a unified multilevel framework using EHIS Wave 3 data.

### Aims

This study investigated the association between social support and participation in colorectal, breast, and cervical screening across 18 European countries using a multilevel framework. It aimed to: (1) assess whether there is an association between social support and participation in cancer screening across all three types, (2) determine whether there is an association between country-level factors and cancer screening participation, (3) estimate between-country variation in cancer screening participation rates, and to examine how much of this variation can be explained by country-level factors.

## Methods

### Study design and study population

EHIS is a population-based survey that gathered information on the general health status of the population, their use and unmet needs of health care services, and their lifestyle or health-related behaviours. Conducted between 2018 and 2020, EHIS Wave 3 covered individuals aged 15 and above living in private households across 32 countries – which are the 27 EU member states plus Iceland, Norway, Serbia, Albania, and Türkiye [21]. This study excluded 14 of these countries due to the following reasons. Data for three countries (Albania, France, and Türkiye) were not released publicly, and nine countries (Belgium, Bulgaria, Croatia, Cyprus, Czechia, Italy, Poland, Romania, and Serbia) had surveyed multiple people per household. As healthcare utilisation and general health status tends to cluster strongly within households [22], these countries were excluded to ensure a comparable two-level analysis across the remaining countries included in this study. Ireland was excluded from the analysis due to data quality issues related to the key study variables of household income and employment status, as illustrated in Supplementary Fig. 1 [F1]. Among those aged 50–74, Ireland showed the highest proportion of missing household income (44%) and atypical employment classifications, with 39% listed as students and 11% in compulsory military or civil service, far above the ≤ 0.5% and ≤ 0.1% observed elsewhere. Mental health was identified as a potential confounding factor; therefore, Spain was excluded as data on this variable were not made available. Variable construction is described below.

### Outcome variable

**Fig. 1:**
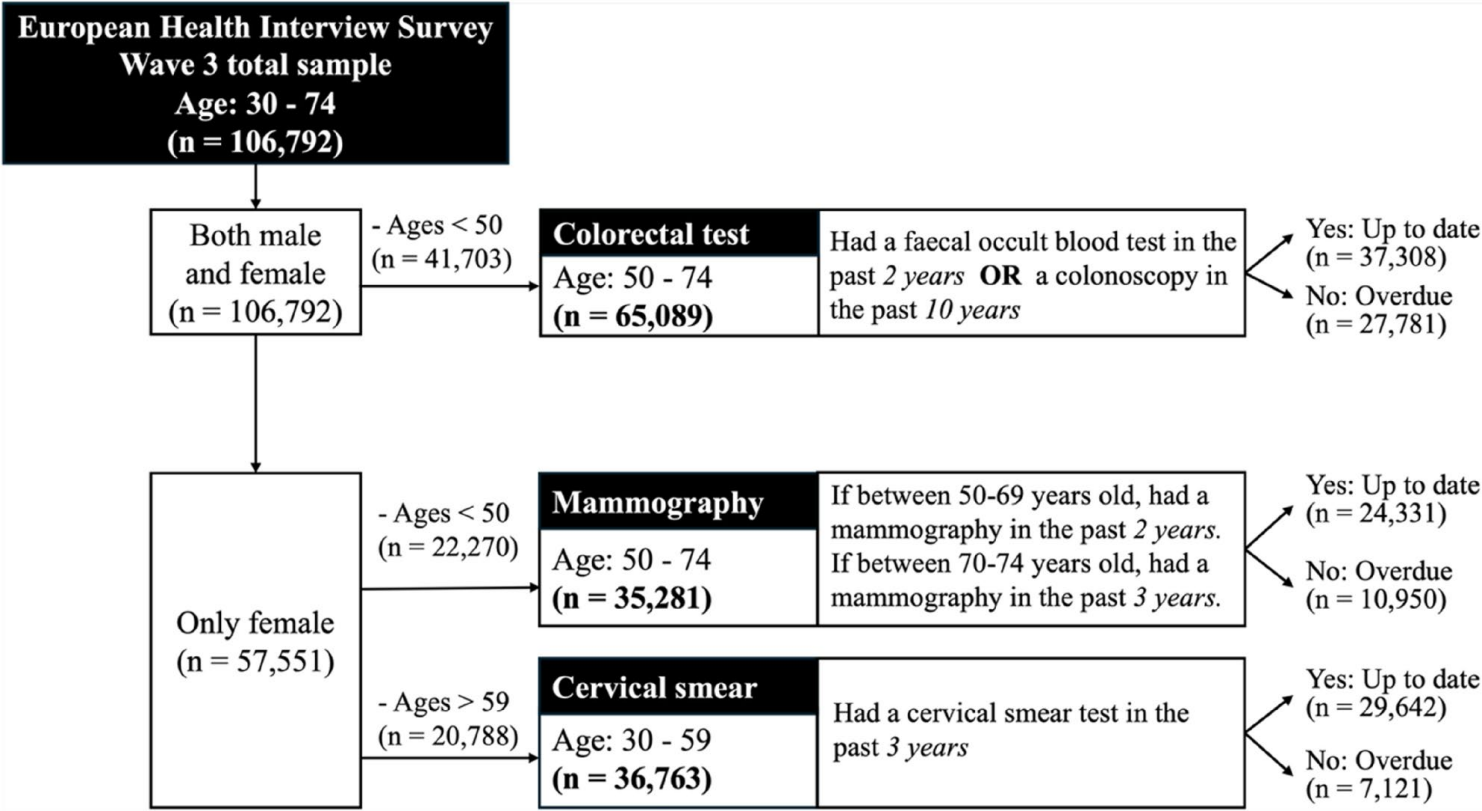
Flowchart of participant inclusion and exclusion for colorectal, breast, and cervical cancer screening outcomes in 18 European countries (2018–2020)

The outcomes of colorectal tests, mammography, and cervical smear tests were analysed as separate outcomes due to differences in target populations (Fig. 1). Target age groups and intervals between screenings vary across countries’ cancer screening programmes [2], hence general guidelines from the European Colorectal Cancer Screening Guidelines Working Group [23], European Commission [24], and IARC [2] were used to determine if individuals were “overdue” for cancer screening (Supplementary Table 1 [T1]).

Colorectal screening was recommended for both males and females aged 50–74 years old. Individuals who had not undergone a faecal occult blood test in the past two years or a colonoscopy in the past ten years were considered overdue [10, 23]. Mammography was recommended every two years for females aged 50–69 and every three years for those aged 70–74 [24]. Accordingly, females in these age groups were considered overdue for breast cancer screening if they had not undergone a mammogram within the recommended interval. Cervical screening guidelines have evolved over time, shifting from Pap smears for females aged 20–30 [25] to Human Papillomavirus (HPV) testing as the preferred method [26]. However, as the EHIS did not collect information on HPV testing [27], this study defined cervical cancer screening only in terms of Pap smears. Screening was recommended every three years [2], and in line with IARC guidelines, this study focused on females aged 30–59 [2].

### Individual-level independent variable

Social support was defined as an individual’s perceived availability of people who can be trusted and who make the individual feel cared for, loved, esteemed, and valued [28]. The extent of an individual’s social support was measured through the Oslo-3 Social Support Scale (OSS-3) listed in Supplementary Fig. 1 [F1] (see response options in Supplementary Table 1 [T1]). Raw scores were given for each item, and when summed, ranged between 3 and 14. A total score from 3 to 8 was classified as poor support, 9 to 11 as intermediate support, and 12–14 as strong support [28–29].

### Country-level independent variables

‘Population-based organised programmes’ are those in which eligible individuals are systematically identified and personally invited to participate, with implementation and monitoring overseen by strong central management and procedures based on firm evidence [2]. Similar to previous research [18], the availability of cancer screening programmes were classified as follows: ‘none’ for the absence of a programme or for non-population-based programmes; ‘partial’ for population-based programmes that were not fully implemented (i.e., in planning, pilot, or roll-out phases, or limited to specific regions); and ‘organised’ for fully implemented, nationwide, population-based programmes. Data was obtained from the cancer screening implementation report by IARC [2]. Additionally, data for Iceland and Norway were supplemented with colorectal screening programme availability from Cardoso et al. [30], and cervical and breast screening programme availability from Jolidon et al. [18] (Supplementary Table 2 [T2]).

Three additional country-level variables were investigated, including SPI [19] household out-of-pocket payments for healthcare as a percentage of total current health expenditure [31], and GP density per 100,000 inhabitants [32]. As a majority of EHIS data were collected in 2019, country-level indicators were obtained for that year where available (see Supplementary Table 1 [T1] for exceptions).

### Individual-level potential confounders

This study controlled for age, sex, employment status, and education level, the latter categorised according to the International Standard Classification of Education [33]. It also adjusted for household type (couple without a child, couple with a child, alone, lone parent with child, and others), need and affordability of medical care in the past twelve months (needed and could afford care, needed but could not afford care, and did not need care), degree of urbanisation (cities, towns and suburbs, and rural areas), and household income quintile. This study used the pre-coded household income quintile variable in the data which was derived from the total net monthly income of a household and equalised to reflect differences in a household’s size and composition [27]. Perceived and evaluated needs were controlled for through self-perceived general health, long-standing health problems, limitations in activities due to health problems, and indicators of poor mental health. Indicators of poor mental health were measured through the Patient Health Questionnaire eight-item depression scale (PHQ-8) [34] in which scores were added to produce a total score between 0 and 24 points. Total scores were then classified as follows: ‘none’ (0–4), ‘mild’ (5–9), ‘moderate’ (10–14), ‘moderately severe’ (15–19), and ‘severe’ (20–24).

The presence of comorbidities was derived from a series of 16 questions which asked whether respondents have or had a particular chronic condition in the past 12 months (Supplementary Table 1 [T1]). The Netherlands lacked information on high blood lipids. However, given that high blood lipids are often found in conjunction with other chronic diseases [35], and that the responses were eventually aggregated into three categories (none, one condition, and two or more conditions), the Netherlands was retained in the analysis despite this omission.

### Data preparation

Among the total sample of individuals aged 30–74 across 18 countries (*n* = 106,792), 80.8% (*n* = 86,268) had complete data for all selected variables in this study. Additionally, 13.3% (*n* = 14,234) had missing data for one variable, while 2.3% (*n* = 2,469) had missing data for two variables. The remaining 3.5% (*n* = 3,721) had missing data for three or more variables. Of the chosen variables from EHIS, only sex and age had complete data; all other variables exhibited varying degrees of missingness (see Supplementary Table 3 [T3]). To address this, a total of 21 imputed data frames were generated using the ‘mice’ [36] function in R. These imputed datasets were then merged into a single data frame using the ‘merge imputations’ function from the ‘sjmisc’ [37] package. All other analyses were conducted in Stata 18 [38] and all subsequent analyses were performed using the imputed data.

### Statistical analyses

This study used two-level multilevel logistic models, with individuals at level one nested within countries in level two. First, a null two-level model was built with only the intercept ($\:{\beta\:}_{0})$ and country residual ($\:{u}_{0j})$ to determine if cancer screening participation differed significantly across countries. Model 1 was a random intercept model which included only the individual-level explanatory variable of social support levels (*ss*). In Model 2, the individual-level potentially confounding variables were added, all of which were categorical. These included: age, sex, employment status (*emp*), education level (*ed*), household type (*hht*), need and affordability of medical care (*aff*), degree of urbanisation (*ur*), household income (*hhi*), perceived general health (*genh*), long-standing health problems (*long*), limitations in activities (*lim*), indicators of poor mental health (*mh*), and presence of comorbidities (*com*). In Model 3, the categorical country-level factor was added: availability of the screening program (*prog*). The numerical country-level factors were included too: Social Progress Index (*spi*), household out-of-pocket payments as a percent of total health care expenditure (*hhoop*), and density of general practitioners per 100,000 inhabitants (*gp*).

Model 3 is written as:

$$\begin{aligned}\mathrm{log}\left(\frac{{\pi\:}_{ij}}{1-\:{\pi\:}_{ij}}\right)=&{\beta\:}_{0}+{\beta\:}_{1}{ss}_{ij}+{\beta\:}_{2}{age}_{ij}+{\beta\:}_{3}{sex}_{ij}\\&+{\beta\:}_{4}{emp}_{ij}+{\beta\:}_{5}{ed}_{ij}+{\beta\:}_{6}{hht}_{ij}+\\&{\beta\:}_{7}{aff}_{ij}+{\beta\:}_{8}{ur}_{ij}+{\beta\:}_{9}{hhi}_{ij}\\&+{\beta\:}_{10}{genh}_{ij}+{\beta\:}_{11}{long}_{ij}+{\beta\:}_{12}{lim}_{ij}+\\&{\beta\:}_{13}{mh}_{ij}+{\beta\:}_{14}{com}_{ij}+{\beta\:}_{15}prog\\&+{\beta\:}_{16}{spi}_{j}+{\beta\:}_{17}{hhoop}_{j}+{\beta\:}_{18}{gp}_{j}+{u}_{0j}\end{aligned}$$

where.

- i denotes the individual (level one).
- j denotes the country (level two).
- log[π_ij/(1-π_ij)] is the log-odds of being overdue for cancer screening versus up to date for individual i in country j.

Crude and adjusted odds ratios (ORs and aORs), along with their 95% confidence intervals (CIs), were reported for fixed effects. Country-level variation was quantified using the intraclass correlation coefficient (ICC) and the median odds ratio (MOR). The impact of each country-level variable on cancer screening uptake relative to country-level variance was assessed using the 80% interval odds ratio (IOR-80). The ICC, MOR, and IOR-80 were calculated following the definitions and formulas outlined by Merlo et al. [39]; with the ICC specifically derived using the linear threshold model approach. Predicted probabilities from Model 3 were estimated using the margins command. Statistical significance was defined as *p* < 0.05.

## Results

Table 1 presents the main characteristics of the study population, stratified by sex. A slight majority of participants (53.9%) were female, and the proportion of respondents was somewhat higher among those aged 50 to 69 years. Most participants had medium to high education levels (80%), were employed (61%), and could afford medical care when needed (69%). The largest share of respondents (16%) came from Germany, while the smallest came from Iceland (2.5%).

**Table 1:** Descriptive characteristics of the study population by sex in 18 European countries (2018–2020)

	Male	Female		Male	Female
	*N* = 49,241 (100%)	*N* = 57,551 (100%)		*N* = 49,241 (100%)	*N* = 57,551 (100%)
Age groups			Indicators of poor mental health		
30–34	4,340 (8.8%)	4,927 (8.6%)	None	40,634 (82.5%)	43,342 (75.3%)
35–39	4,745 (9.6%)	5,354 (9.3%)	Mild	6,335 (12.9%)	10,139 (17.6%)
40–44	5,083 (10.3%)	5,891 (10.2%)	Moderate	1,462 (3.0%)	2,701 (4.7%)
45–49	5,265 (10.7%)	6,098 (10.6%)	Moderately Severe	571 (1.2%)	983 (1.7%)
50–54	5,960 (12.1%)	7,141 (12.4%)	Severe	239 (0.5%)	386 (0.7%)
55–59	6,357 (12.9%)	7,352 (12.8%)	Presence of comorbidities		
60–64	6,229 (12.7%)	7,474 (13.0%)	None	17,402 (35.3%)	17,919 (31.1%)
65–69	6,088 (12.4%)	7,047 (12.2%)	1 Condition	12,494 (25.4%)	13,186 (22.9%)
70–74	5,174 (10.5%)	6,267 (10.9%)	2 + Conditions	19,345 (39.3%)	26,446 (46.0%)
Education level			Self-perceived general health		
High	19,600 (39.8%)	23,397 (40.7%)	Very good	10,541 (21.4%)	11,566 (20.1%)
Medium	20,139 (40.9%)	22,354 (38.8%)	Good	23,423 (47.6%)	25,980 (45.1%)
Low	9,502 (19.3%)	11,800 (20.5%)	Fair	11,756 (23.9%)	15,375 (26.7%)
Employment status			Bad	2,876 (5.8%)	3,857 (6.7%)
Employed	32,139 (65.3%)	32,856 (57.1%)	Very bad	645 (1.3%)	773 (1.3%)
Retired	12,546 (25.5%)	14,639 (25.4%)	Long-standing health problem		
Unemployed	2,083 (4.2%)	2,551 (4.4%)	No	25,822 (52.4%)	27,022 (47.0%)
Other	2,473 (5.0%)	7,505 (13.0%)	Yes	23,419 (47.6%)	30,529 (53.0%)
Household type			Limitations in activities		
Couple w/o child	16,811 (34.1%)	18,051 (31.4%)	No	35,841 (72.8%)	39,644 (68.9%)
Couple & ≥ 25 year child	3,154 (6.4%)	3,054 (5.3%)	Limited-not severely	10,256 (20.8%)	14,100 (24.5%)
Couple & <25 year child	15,082 (30.6%)	16,406 (28.5%)	Severely limited	3,144 (6.4%)	3,807 (6.6%)
Lone parent & <25 year child	787 (1.6%)	3,299 (5.7%)	Countries		
Alone	9,651 (19.6%)	12,027 (20.9%)	AT (Austria)	5,018 (10.2%)	5,713 (9.9%)
Other	3,756 (7.6%)	4,714 (8.2%)	DE (Germany)	8,019 (16.3%)	9,039 (15.7%)
Need and affordability of medical care			DK (Denmark)	2,071 (4.2%)	2,704 (4.7%)
Needed and could afford care	32,830 (66.7%)	40,627 (70.6%)	EE (Estonia)	1,467 (3.0%)	1,973 (3.4%)
Needed but could not afford care	2,081 (4.2%)	3,306 (5.7%)	EL (Greece)	2,634 (5.3%)	2,941 (5.1%)
Did not need care	14,330 (29.1%)	13,618 (23.7%)	FI (Finland)	1,973 (4.0%)	2,551 (4.4%)
Household Income			HU (Hungary)	1,891 (3.8%)	2,147 (3.7%)
5th Quintile (Highest)	12,337 (25.1%)	11,524 (20.0%)	IS (Iceland)	1,269 (2.6%)	1,418 (2.5%)
4th Quintile	11,814 (24.0%)	12,752 (22.2%)	LT (Lithuania)	1,362 (2.8%)	1,998 (3.5%)
3rd Quintile	10,991 (22.3%)	12,703 (22.1%)	LU (Luxembourg)	1,661 (3.4%)	1,845 (3.2%)
2nd Quintile	7,957 (16.2%)	11,458 (19.9%)	LV (Latvia)	1,706 (3.5%)	2,377 (4.1%)
1st Quintile (Lowest)	6,142 (12.5%)	9,114 (15.8%)	MT (Malta)	1,526 (3.1%)	1,660 (2.9%)
Degree of urbanisation			NL (Netherlands)	2,841 (5.8%)	2,948 (5.1%)
Cities	16,712 (33.9%)	20,226 (35.1%)	NO (Norway)	2,808 (5.7%)	2,780 (4.8%)
Towns & suburbs	16,752 (34.0%)	19,455 (33.8%)	PT (Portugal)	4,624 (9.4%)	5,828 (10.1%)
Rural	15,777 (32.0%)	17,870 (31.1%)	SE (Sweden)	3,323 (6.7%)	3,257 (5.7%)
			SI (Slovenia)	3,258 (6.6%)	3,827 (6.6%)
			SK (Slovakia)	1,790 (3.6%)	2,545 (4.4%)

Overall, 71% of respondents reported no limitations in daily activities due to health problems, whereas just over half (51%) reported having a long-standing health problem, with a higher proportion among females (53%) than males (48%). Approximately 79% of participants had no indicators of poor mental health. The most common household types were couples living without a child (33%), couples living with a child younger than 25 years old (30%) and individuals living alone (20%). A higher proportion of females (5.7%) than males (1.6%) lived as lone parents with a child younger than 25 years.

**Fig. 2:**
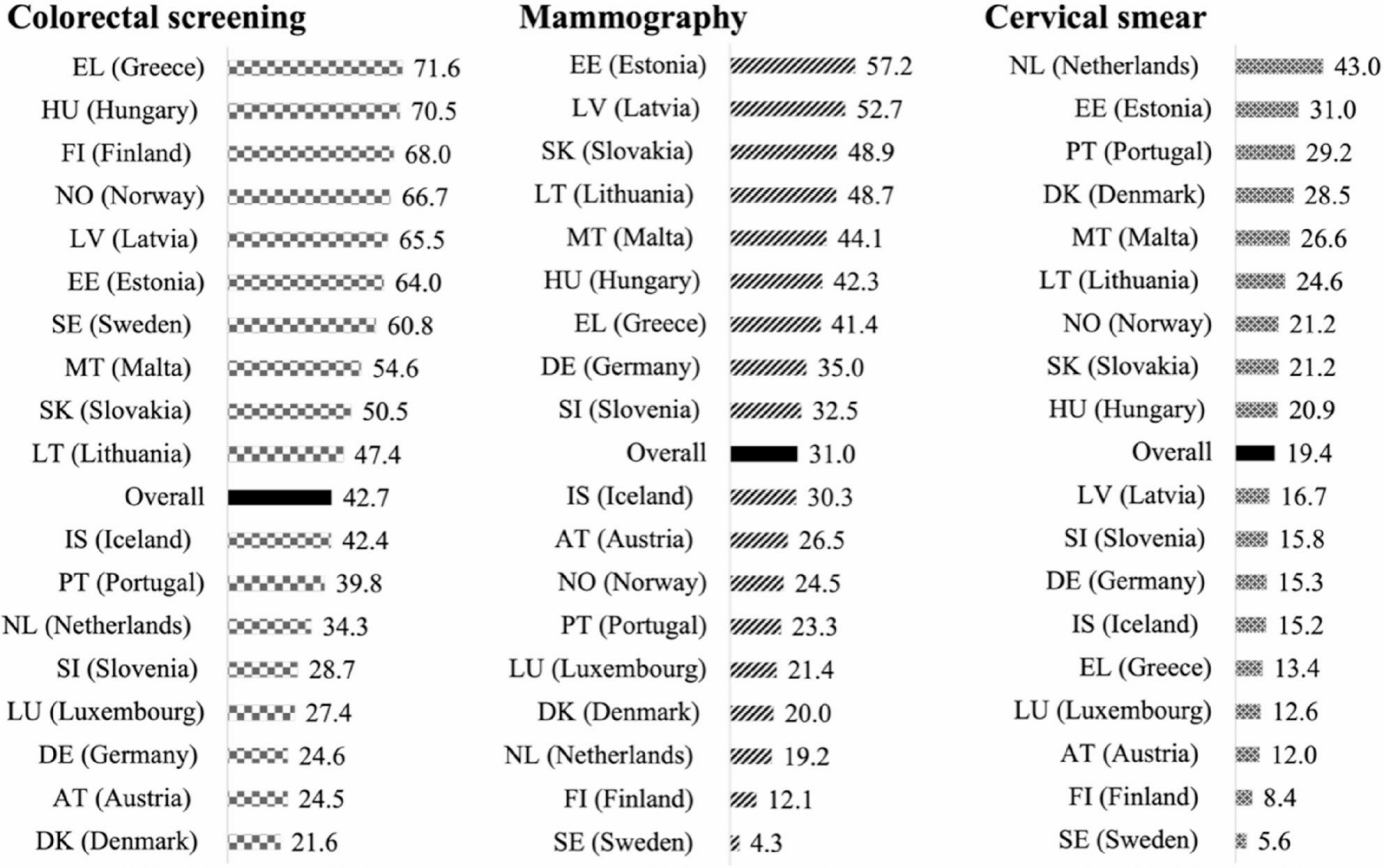
Country-specific and overall prevalence of individuals overdue for colorectal, breast, and cervical cancer screening in 18 European countries (2018–2020)

The overall prevalence of being overdue for screening was 43% for colorectal, 31% for breast, and 19% for cervical tests. Across countries, this ranged from 22% in Denmark to 72% in Greece for colorectal screening, 4% in Sweden to 57% in Estonia for mammography, and 6% in Sweden to 43% in the Netherlands for cervical smear tests (Fig. 2 and Supplementary Table 4 [T4]).

### Fixed-effect analyses

**Fig. 3:**
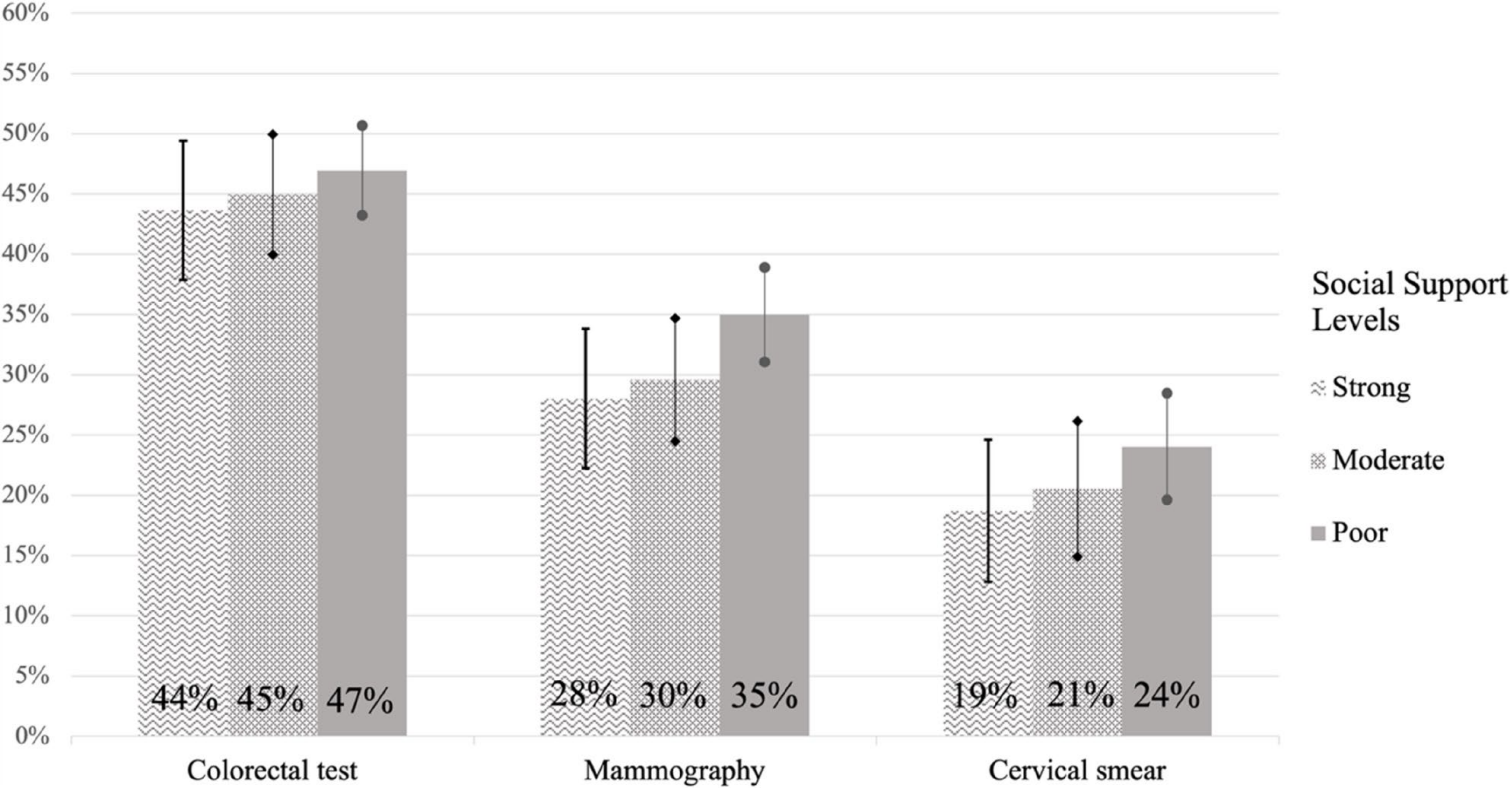
Predicted probabilities (%) of being overdue for colorectal, breast, and cervical cancer screening from the adjusted multilevel logistic regression model by social support levels in 18 European countries (2018–2020)

Model 1 in Table 2 presents crude odds ratios for the association between social support levels and cancer screening uptake. After adjustment for individual-level confounders, Model 2 shows that individuals with moderate or poor social support had higher odds of being overdue for all types of cancer screening compared with those with strong social support. Particularly for individuals with poor social support, the association was strongest for breast cancer screening (aOR 1.45; 95%CI: 1.35–1.57), and cervical cancer screening (aOR 1.42; 95%CI: 1.30–1.56), and weaker for colorectal cancer screenings (aOR 1.17; 95%CI: 1.11–1.24). Further adjustment for country-level factors in Model 3 did not alter the results, with aOR and 95%CI remaining consistent with those in Model 2. The predicted probability of being overdue for breast cancer screening (Fig. 3) was 28% among individuals with strong social support, 30% with moderate support, and 35% with poor support.

**Table 2:** Associations of social support and country-level factors with the odds of being overdue for colorectal, breast, and cervical cancer screening across 18 European countries (2018–2020)

	Colorectal test	Mammography	Cervical smear
	(*n* = 65,089)	(*n* = 35,281)	(*n* = 36,763)
	OR/aOR (95% CI) [IOR-80]	OR/aOR (95% CI) [IOR-80]	OR/aOR (95% CI) [IOR-80]
Null Model (Intercept only)			
ICC (%)	14.5 (8.1–24.6)	16.6 (9.3–27.8)	9.6 (5.2–17.2)
MOR	2.04 (1.56–2.51)	2.16 (1.61–2.71)	1.76 (1.43–2.09)
Model 1			
Social support level (ref: strong)			
Moderate	1.04 (1.00–1.08)	1.11*** (1.05–1.17)	1.24*** (1.16–1.31)
Poor	1.11*** (1.06–1.18)	1.58*** (1.47–1.70)	1.79*** (1.65–1.95)
ICC (%)	14.5 (8.1–24.6)	16.4 (9.2–27.6)	9.7 (5.3–17.3)
MOR	2.04 (1.56–2.51)	2.15 (1.61–2.70)	1.76 (1.43–2.10)
Model 2			
Social support level (ref: strong)			
Moderate	1.07** (1.02–1.11)	1.09** (1.03–1.15)	1.14*** (1.07–1.21)
Poor	1.17*** (1.11–1.24)	1.45*** (1.35–1.57)	1.42*** (1.30–1.56)
ICC (%)	16.8 (9.5–28.0)	16.5 (9.2–27.6)	9.6 (5.2–17.1)
MOR	2.18 (1.62–2.73)	2.16 (1.61–2.70)	1.76 (1.43–2.09)
Model 3			
Social support level (ref: strong)			
Moderate	1.07** (1.02–1.11)	1.09** (1.03–1.15)	1.14*** (1.07–1.21)
Poor	1.17*** (1.11–1.24)	1.45*** (1.35–1.57)	1.42*** (1.30–1.56)
*Country-level factors* Program availability (ref: organised)			
Partial	2.65* (1.04–6.77) [0.94–7.47]	1.048 (0.45–2.42) [0.37-3.00]	0.946 (0.47–1.92) [0.37–2.43]
None	3.17* (1.16–8.67) [1.13–8.95]	0.741(0.26–2.10) [0.26–2.12]	0.535 (0.26–1.10) [0.21–1.37]
Social Progression Index	1.03 (0.93–1.15) [0.37–2.91]	0.890 (0.79–1.01) [0.31–2.55]	0.960 (0.86–1.07) [0.37–2.46]
Household out-of-pocket payments	1.05 (1.00–1.10) [0.37–2.95]	1.006 (0.96–1.06) [0.35–2.88]	0.987 (0.94–1.03) [0.39–2.53]
GPs per 100,000 inhabitants	0.99 (0.99–1.00) [0.35–2.80]	0.997 (0.99- 1.00) [0.35–2.85]	1.003 (0.99–1.01) [0.39–2.57]
ICC (%)	9.0 (4.9–16.1)	9.3 (5.0– 16.6)	7.6 (4.0–13.8)
MOR	1.73 (1.42–2.03)	1.74 (1.42– 2.06)	1.64 (1.37–1.91)

The null model has no explanatory variable. Subsequent models build upon it and each other by adding more variables

Model 1 included social support levels. Model 2 adjusted for potential confounders: age, sex, employment status, education level, household type, need and affordability of medical care, degree of urbanisation, household income, perceived general health, perceived long-standing health problem, perceived limitations in activities, indicators of poor mental health, and presence of comorbidities. Model 3 added country-level factors of availability of population-based screening programmes, social progression index, household out-of-pocket payments % share of total current health expenditure, and general practitioners (GPs) per 100,000 inhabitants

Odds Ratio; *CI* Confidence Interval, *IOR-80* Interval Odds Ratio at 80%, *ICC* Interclass Correlation, *MOR* median odds ratio

*** *p* < 0.001, ** *p* < 0.01, * *p* < 0.05

At the country-level, the lack of an organised, population-based country screening programme was associated with higher odds of being overdue for colorectal tests, whereas no such association was observed for breast or cervical tests. Individuals residing in countries without organised colorectal screening programme had higher odds of being overdue for screening (OR 3.17; 95%CI: 1.16–8.67) compared with those in countries with organised programmes. Other associations between country-level factors (i.e., SPI, household out-of-pocket payments, and GP density) and screening uptake were not observed. Notably, the only IOR-80 interval excluding one [1.13–8.95] corresponded to the absence of an organised colorectal screening programme, indicating that this was the only country-level variable showing a strong and consistent association relative to the remaining residual between country heterogeneity.

### Random effect analyses

**Fig. 4:**
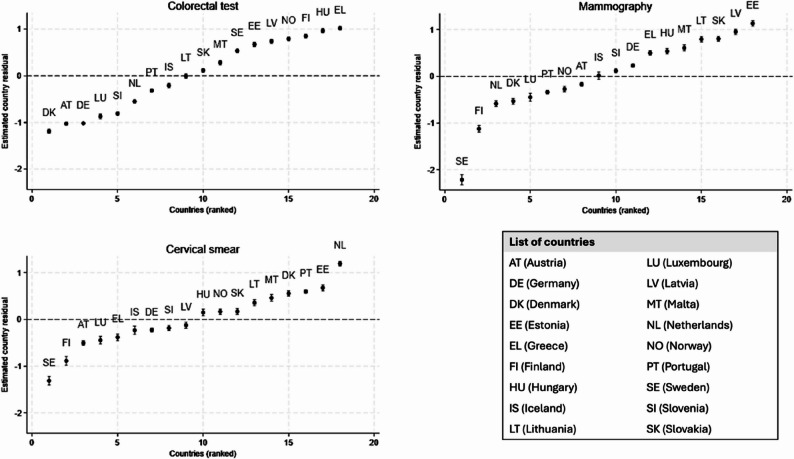
Country-level variation in the prevalence of being overdue for colorectal, breast, and cervical cancer screening: null model estimates across 18 European countries (2018–2020). The dotted line at 0 represents the overall intercept, which is the mean log-odds of being overdue across all countries. Countries whose residuals and 95% confidence intervals do not intersect the overall intercept differ significantly from the overall average, with values below 0 indicating lower log-odds and values above 0 indicating higher log-odds of being overdue for screening

The null model (Fig. 4) indicates substantial between-country variation in the prevalence of being overdue for cancer screening. Nearly all 95% confidence intervals for the country-specific intercepts did not overlap with the overall mean intercept (shown by the dotted line at zero), confirming notable between-country differences and supporting the use of a multilevel approach. The ICCs of the null model were 16.6% (95%CI: 9.3–27.8) for mammography, 14.5% (95%CI: 8.1–24.6) for colorectal screening and 9.6% (95%CI: 5.2–17.2) for cervical cancer screening (Table 2). Likelihood ratio tests further supported the inclusion of the country level for all outcomes (*p* < 0.005). Comparing Model 3 with Model 2 in Table 2 shows that country-level factors explained approximately half of the ICC of colorectal screening (46%) and mammography (44%), but only 21% for cervical cancer screening.

## Discussion

### The role of social support in screening uptake

Individuals with poor social support were more likely to be overdue for cancer screenings which is consistent with findings from prior studies across multiple European countries [6, 14]. This study strengthens the existing evidence by including data from multiple European countries and accounting for clustering through multilevel modelling. A recent systematic review reported psychological factors, such as embarrassment, shame, discomfort, and fear, especially of being diagnosed with cancer, as some of the most frequently cited barriers to screening uptake [4]. Social support may serve as an enabling resource to help overcome these barriers by facilitating information sharing, promoting self-care through the sense of meaning and belonging that individuals derive from their roles within social networks, and reducing anxiety through emotional support [6]. While identifying individuals with poor social support in society may be challenging, healthcare systems can help mitigate its effects by providing patient navigation and outreach services. Such services, which facilitate comprehensive support such as building relationships, addressing barriers, providing educational materials, and coaching patients to engage with providers, have shown great potential in improving participation in cancer screening services [40].

Our study shows that social support had a stronger association with participation in breast and cervical cancer screening than in colorectal screening across the European population. This finding was somewhat unexpected, as the colorectal sample included males and previous research found stronger effects of social support among males than females in countries without organised colorectal screening [14]. Additional barriers unique to colorectal screening, such as feelings of disgust [4], physical and psychological discomfort from invasive colonoscopy, and the burden of bowel preparation through laxative or dietary changes [41] may discourage individuals from discussing their experiences or recommending screening to others. This suggests the need to further explore how the influence of social support varies by screening type. Categorising social support as strong, moderate, and poor helps identify where more notable increases in the risk of being overdue occur, for example in breast cancer screening, where the predicted probability of being overdue rises from 30% among individuals with moderate support to 35% among those with poor support.

### The role of contextual determinants in screening uptake

Consistent with previous research [10], individuals residing in countries with organised colorectal screening programmes were more likely to be up to date, suggesting the facilitating role of such programmes. The lack of an association between having organised screening programmes and uptake of breast and cervical cancer screenings may result from a reduced statistical power as there were fewer countries with no or partial screening programmes for these cancers (Supplementary Table 2 [T2]). Alternatively, it may reflect higher rates of opportunistic testing. Opportunistic testing, which are screenings conducted outside the framework of an organised programme, often fall outside targeted age groups and recommended intervals, and from an equity point of view, tends to exacerbate social inequalities by disproportionately benefiting more advantaged groups, such as those with higher socioeconomic status [2] or residing in less deprived areas [42], which is a process commonly referred to as ‘intervention-generated inequalities’ or IGIs [43]. Although all EU countries conduct opportunistic testing, it is not recommended by the European Council, which considers it relatively inefficient, cost-ineffective, and less advantageous compared to population-based organised programmes. The frequent lack of registration, monitoring, documentation, and systematic quality assurance in opportunistic testing can contribute to overdiagnosis, overtreatment, and increased healthcare costs [2].

It may also be the case that the presence of organised screening programmes leads to higher uptake when supported by other country-level factors. For instance, De Prez et al. [17] that organised cervical cancer screening reduced inequalities in uptake only in countries with a high decommodification index – a composite measure of unemployment benefits, social assistance, and health provision. However, our study did not find associations between SPI, household out-of-pocket payments, and GP density with screening uptake. Higher levels of macro-level gender equality, as measured by the Gender Inequality Index, have previously been associated with lower participation in breast and cervical cancer screening [44]. It is possible that the SPI, which includes gender equality, encompasses too broad a set of indicators, diluting effects specifically related to screening behaviour. Further research is needed to explore whether these country-level factors modify the association between social support and screening participation, as they have previously been shown to influence education- and income-based inequalities in screening participation [17, 18].

### Between-country variation in screening uptake

This study estimates the proportion of variation in cancer screening that is attributable to country-level effects (ICC) across 18 European countries using the most current EHIS data. The findings indicated substantial between-country variation, with ICC of 16.6% for breast, 9.6% for cervical, and 14.5% for colorectal cancer screening. The relatively lower ICC for cervical screening may reflect more widespread opportunistic screening practices, which reduce between-country variability by increasing coverage even in settings without organised programmes [18]. Additionally, cervical screening can be self-administered, unlike mammography or colonoscopy. Although some colorectal tests (e.g., faecal occult blood tests) can also be self-administered, individuals generally express greater confidence in performing cervical self-tests and in the reliability of their results [41].

The country-level effects examined in this study explained a larger proportion of the ICC in colorectal and breast cancer screening than for cervical cancer screening. Similarly, other research [18] found that screening programme availability accounted for little of the ICC in cervical cancer screening, whereas gatekeeping primary care systems, which requires general practitioner referrals, explained a greater share of the variation. Further research is needed to identify additional country-level factors that may account for the remaining differences in screening participation.

### Strengths and limitations

This study is among the few to examine participation in breast, cervical, and colorectal screening in a comparable manner, using the same set of variables from the same survey across analyses. It also provides a robust cross-national analysis by drawing on the most recently available EHIS data and addressing missing data by multiple imputations. The use of multilevel modelling provides valuable insights into both between-country variation in screening participation and the role of country-level factors. Nevertheless, several limitations should be acknowledged. The cross-sectional design precludes causal inference, restricting conclusions to observed associations. Generalisability to the wider European population may be limited, as countries that surveyed multiple individuals per household were excluded. Screening uptake was self-reported, which may introduce recall and social desirability bias. The omission of information on HPV tests in the EHIS dataset may introduce bias in prevalence and correlation estimates as lower participation rates in Pap smear screening may reflect the adoption of HPV testing as an additional screening method in certain countries – although EU guidelines on HPV screening were only published in 2015 [2]. Additionally, the dataset lacked information on the context of screening participation—specifically, whether it occurred within an organised programme or opportunistically. Social support was also self-reported and may be influenced by social desirability bias, potentially leading to overestimation.

The potential impact of the Covid-19 pandemic on these results is possible but likely to be minimal since fieldwork for all countries in this study was completed before February 2020, except for Latvia, Malta, and Germany, which concluded data collection in February, April, and September 2020, respectively [21] and Covid-19 lockdown measures in these three countries were not implemented until March 2020 [45]. Information on the organisation status of screening programmes were taken from other publications [2, 18, 30] that do not coincide exactly with the survey period; therefore, programme status may have differed at the time of data collection.

The heterogenous adoption of target populations and screening intervals across countries (Supplementary Table 2 [T2]) may result in discrepancies between this study’s classification of being overdue and the countries’ classifications. None of the countries examined in this study initiated breast or cervical cancer screening at ages older than the recommended starting thresholds [2, 46, 47]. However, several countries stop screening at younger ages than those recommended. Specifically, Estonia and Hungary limited breast cancer screening to individuals up to 64 years old rather than 69 and Malta’s pilot cervical cancer screening programme targeted only individuals aged between 25 and 35 [2]. By contrast, more national programmes for colorectal cancer screening deviate from the recommended age range. Screening is initiated at 55 years in Luxembourg and Malta and at 60 years in Estonia, Finland, and Sweden, while screening ends at 66 years in Malta, 69 years in Estonia, Finland, and Sweden, and 70 years in Portugal, Greece, and Hungary [2]. While some countries employed shorter screening intervals than recommended, none applied longer intervals for breast cancer screening [2, 47]. The same characteristic was observed for colorectal cancer screening programmes [2]. In contrast, for cervical cancer screening, several countries used longer intervals of five years (Estonia, Finland, and the Netherlands), while others such as Denmark and Sweden [2] and Norway [46] applied age-differentiated intervals, with three-year intervals for younger age groups and five-year intervals for older age groups.

## Conclusions

Social support is a key factor to consider when promoting participation in breast, cervical, and colorectal cancer screening. Its association was stronger for breast and cervical screening than for colorectal screening. The presence of organised population-based screening programs was associated with higher participation rates for colorectal cancer screening. At the country level, screening programme availability, social progress, household out-of-pocket payments, and general practitioner density collectively explained nearly half of the variation in breast and colorectal cancer screening uptake, but a smaller proportion of the variation in cervical cancer screening.

## Supplementary Information

Supplementary Material 1.

## Data Availability

The EHIS data supporting the conclusions of this article are available upon request in the Eurostat repository, https://ec.europa.eu/eurostat/web/microdata/european-health-interview-survey.

## Abbreviations

aOR: Adjusted odds ratio
CI: Confidence interval
EHIS: European health interview survey
EU: European union
GP: General practitioner
HPV: Human papillomavirus
IARC: International agency for research on cancer
ICC: Intraclass correlation coefficient
IGI: Intervention-generated inequalities
IOR-80: 80% Interval odds ratio
MOR: Median odds ratio
OR: Odds ratio
OSS-3: Oslo-3 social support scale
PHQ-8: Patient health questionnaire eight-item depression scale
SPI: Social progress index
