# Supplementary material for: Social support and country-level factors associated with cancer screening participation in 18 European countries: a multilevel analysis of the European Health Interview Survey
Source: Arch Public Health. 2026 Aug 6;84:178. doi: 10.1186/s13690-026-02023-w (PMC13450511; doi:10.1186/s13690-026-02023-w)
Supplement: Supplementary file 1 [file 13690_2026_2023_MOESM1_ESM.docx]

Supplementary Material

| Article title | Social support and country-level factors associated with cancer screening participation in 18 European countries: a multilevel analysis of the European Health Interview Survey |
| --- | --- |
| Journal | Archives of Public Health |
| Author names | Maelodee Chong Armstrong, Mattias Johansson, Megumi Rosenberg, Nawi Ng |
| Corresponding author’s affiliation and e-mail address | School of Public Health and Community Medicine, Institution of Medicine, Sahlgrenska Academy, University of Gothenburg, Sweden. *E-mail address:* [*maelodee.chong@gu.se*](mailto:maelodee.chong@gu.se)*.* |

Supplementary Figure 1. Conceptual framework illustrating the multilevel construct and variables included in this study.

Supplementary Table 1. Variable and coding list

| **Question Code and (Variable Name)** | **Original Question** | **Original Responses or Sources (if not part of EHIS data)** | **Recoded Responses** |
| --- | --- | --- | --- |
| **Outcome variable: Cancer screening participation - colorectal** | | | |
| PA5 | When was the last time you had a faecal occult blood test? | 1 Within the past 12 months  2 1 to less than 2 years  3 2 to less than 3 years  4 3 years or more  5 Never  -1 Not stated  -3 Proxy | For individuals between **50-74**.  If -2 = -2 (not applicable)  If -1,-3 = . (missing)  If 1,2 = 0 (Up to date)  If 3, 4, 5 = 1 (Overdue)  According to [European guidelines](https://www.ncbi.nlm.nih.gov/pmc/articles/PMC5757117/). |
| PA6 | When was the last time you had a colonoscopy? | 1 Within the past 12 months  2 1 to less than 5 years  3 5 to less than 10 years  4 10 years or more  5 Never  -1 Not stated  -3 Proxy | Recommended as [follow-up](https://eur-lex.europa.eu/EN/legal-content/summary/promoting-cancer-screening-in-the-european-union.html) to faecal blood test in individuals between **50-74**. (every 10 – 20 years). Timeframe also European guidelines found [this paper](https://www.ncbi.nlm.nih.gov/pmc/articles/PMC5757117/).  If -2 = -2 (not applicable)  If -1,-3 = . (Missing)  If 1,2,3,4 = 0 (Up to date)  If 5 = 1 (Overdue) |
| Generated variable: (Colorectal cancer screening participation) |  |  | Colorectal screening participation was considered overdue if individual did not have either faecal occult blood test in the past 2 years or colonoscopy in the past 10 years  If faecal occult blood test and colonoscopy = -2 = -2 (missing)  If faecal occult blood test = 0 or/and colonoscopy = 0 = 0 (Up to date)  If faecal occult blood test = 1 and colonoscopy = 1 = 1 (Overdue) |
| **Outcome variable: Cancer screening participation - breast** | | | |
| PA7 (Breast cancer screening participation) | When was the last time you had a mammography (breast X-ray)? | 1 Within the past 12 months  2 1 to less than 2 years  3 2 to less than 3 years  4 3 years or more  5 Never  -1 Not stated  - 2 not applicable  -3 Proxy | Females between **50-69** years old are recommended **biannual screening** and between **70-74** years old recommended **triannual screening**   - If -1,-3 = . (Missing) - If -2 = -2 (Not applicable)   If 50-69…   - If 1,2 = 0 (Up to date) - If 3,4,5 = 1 (Overdue)   If 70-74…   - If 1,2,3 = 0 (Up to date) - If 4,5 = 1 (Overdue)   Guidelines from the [European Commission](https://healthcare-quality.jrc.ec.europa.eu/en/ecibc/european-breast-cancer-guidelines?topic=63&usertype=60&filter_1=96&filter_2=92&updatef2=0). |
| **Outcome variable: Cancer screening participation - cervical** | | | |
| PA8 (Cervical cancer screening participation) | When was the last time you had a cervical smear test? | 1 Within the past 12 months  2 1 to less than 2 years  3 2 to less than 3 years  4 3 years or more  5 Never  -1 Not stated  - 2 not applicable  -3 Proxy | For females between **30-59**  -1 -3 = . (Missing)  -2 = -2 (not applicable)  If 1,2,3 = 0 (Up to date)  If 4,5, = 1 (Overdue)  Pap smear recommended screening interval of 3 years. (Ponti et al., 2017, Jolidon, Bracke, and Burton-Jeangros, 2021).  Human papillomavirus (HPV) was not included. |
| **Exposure variable (at level 1 – individuals): Social support levels** | | | |
| SS1 | How many people are so close to you that you can count on them if you have serious personal problems? | 1. None  2. 1 or 2  3. 3 to 5  4. 6 or more  -1. Not stated  -3. Proxy | 1 = 1  2 = 2  3 = 3  4 = 4 |
| SS2 | How much concern do people show in what you are doing? | 1. A lot of concern and interest  2. Some concern and interest  3. Uncertain  4. Little concern and interest  5. No concern and interest  -1. Not stated  -3. Proxy | 1 = 5  2 = 4  3 = 3  4 = 2  5 = 1 |
| SS3 | How easy is it to get practical help from neighbours if you should need it? | 1. Very easy  2. Easy  3. Possible  4. Difficult  5. Very difficult  -1. Not stated  -3. Proxy | 1 = 5  2 = 4  3 = 3  4 = 2  5 = 1 |
| Generated variable: (Social Support Levels) |  |  | 12/14 = 1 “Strong”  9/11 = 2 “Moderate”  3/8 = 3 “Poor”  Using the Oslo social support scale (OSSS-3), the sum of the scores will range between 3 to 14.  3–8 poor social support  9–11 moderate social support  12–14 strong social support  References: Kilpeläinen and Aromaa, 2008. Kocalevent et al., 2018. |
| **Exposure variables (at level 2 – countries), see dataset in Supplementary Table 2.** | | | |
| Generated variable: (Country-level colorectal screening programme availability) |  | Obtained from Ponti, et al., 2017 and Cardoso et al., 2020  (This reflects the highest level of screening programme availability across both faecal blood tests and colonoscopy) | 2 = 1 “Population-based organised programme”  1 = 2 “Partial”  0 = 3 “None” |
| Generated variable: (Country-level cervical smear programme availability) |  | Obtained from Ponti, et al., 2017 and Jolidon, Bracke and Burton-Jeangros, 2021 | 2 = 1 “Population-based organised programme”  1 = 2 “Partial”  0 = 3 “None” |
| Generated variable: (Country-level mammography programme availability) |  | Obtained from Ponti, et al., 2017 and Jolidon, Bracke and Burton-Jeangros, 2021 | 2 = 1 “Population-based organised programme”  1 = 2 “Partial”  0 = 3 “None” |
| Generated variable: (Social Progress Index (SPI) in 2019) |  | Obtained from the Social Progress Imperative 2025 |  |
| Generated variable: (Household out-of-pocket payment percentage share of total current health expenditure in 2019) |  | Obtained from Eurostat, 2025 |  |
| Generated variable: (GPs per 100,000 population (in 2019 unless commented otherwise)) |  | Obtained from Eurostat, 2022 and Jolidon, et al, 2021.  Note: Three countries did not have 2019 GP density data available in Eurostat. Data were carried forward from 2018 for Finland and from 2017 for Luxembourg. For Slovakia, no historical data were listed in Eurostat, so information from Jolidon, et al (2021) was used; these was reported to have been measured in 2013. |  |
| **Potential confounders: Demographic variables** | | | |
| AGE (Age) | Age of respondent in completed years at the time of the interview | 5 years age groups with top coding (85 and more): 15-17, 18-19, 20-24, …, 85+ | Recoded to  1 “<30”  2 “30-34”  3 “35-39”  4 “40-44”  5 “45-49”  6 “50-54”  7 “55-59”  8 “60-64”  9 “65-69”  10 “70-74”  11 “>74” |
| SEX (Sex) | Sex of respondent | 1. Male  2. Female | - |
| **Potential confounders:** Social structure | | | |
| MAINSTAT (Employment status) | Main activity status (self-declared) | 10 Employed  20 Unemployed  30 Retired  40 Unable to work due to longstanding health problems  50 Student, pupil  60 Fulfilling domestic tasks  70 Compulsory military or civilian service  80 Other  -1 Not stated | -1 = . (missing)  10=1 (Employed)  30=2 (Retired)  20=3 (Unemployed)  40/80=4 (Other) |
| HATLEVEL (Education level) | Educational attainment level  (highest level of education completed) | Based on ISCED classification  0 No formal education or below ISCED 1  1 ISCED 1 Primary education  2 ISCED 2 Lower secondary education  3 ISCED 3 Upper secondary education  4 ISCED 4 Post-secondary non-tertiary education  5 ISCED 5 Short-cycle tertiary education  6 ISCED Bachelor’s or equivalent level  7 ISCED Master’s or equivalent level  8 ISCED Doctoral or equivalent level  -1 Not stated | -1 = missing  If 5,6,7,8 = 1 high education  If 3, 4 = 2 medium education  If 0,1,2 = 3 low education  Following the [International Standard Classification of Education 2011](https://ec.europa.eu/eurostat/statistics-explained/index.php?title=International_Standard_Classification_of_Education_(ISCED)#Background) |
| HHTYPE (Household type) | Type of household | Regrouped answer categories:  1. One-person household (code 10)  2. Lone parent with at least one child aged less than 25 (code 21)  3. Couple without any children (code 31)  4. Couple with at least one child aged less than 25 (code 32)  5. Couple all children aged 25 or more (code 33)  6. Other (codes 22 and 40)  -1. Not stated | -1=.  3=1 “Couple w/o child”  5=2 “Couple & >25y child”  4=3 “Couple & <25y child”  2=4 “Lone par & <25y child”  1=5 “Alone”  6=6 “Other”  Note: Lone parent with all children older than 25 is grouped together with “other type of household” in Other. |
| **Potential confounders:** Personal/family resources income, urbanisation | | | |
| UN2A (Need and affordability of medical care) | Was there any time in the past 12 months when you needed the following kinds of health care, but could not afford it? … medical care | 1 Yes  2 No  3 No need  -1 Not stated  -3 Proxy | -3/-1=.  2=1 “No”  1=2 “Yes”  3=3 “No need” |
| DEG_URB (Degree of urbanisation) | Degree of urbanisation | 1 Cities  2 Towns and suburbs  3 Rural areas  -1 Not stated | -1=.  1=1 “Cities”  2=2 “Towns and suburbs”  3=3 “Rural areas” |
| HHINCOME (Household income quintile) | Net monthly equivalised income of the household | 1 Below 1^st^ quintile  2 Between 1^st^ and 2^nd^ quintile  3 Between 2^nd^ and 3^rd^ quintile  4 Between 3^rd^ and 4^th^ quintile  5 Between 4^th^ and 5^th^ quintile  -1 Not stated | -1 = . (missing)  5 = 1 (5^th^ Quintile (highest))  4 = 2 (4^th^ Quintile)  3 = 3 (3^rd^ Quintile)  2 = 4 (2^nd^ Quintile)  1 = 5 (1^st^ Quintile (lowest)) |
| **Potential confounders:** Perceived needs | | | |
| HS1 (Self-perceived general health) | How is your health in general? Is it … | 1 Very good  2 Good  3 Fair (neither good nor bad)  4 Bad  5 Very bad  -1 Not stated  -3 Proxy | -3/-1 = .  1=1 “Very good”  2=2 “Good”  3=3 “Fair”  4=4 “Bad”  5=5 “Very bad” |
| HS2 (Long-standing health problems) | Do you have any longstanding illness or health problem? | 1 Yes  2 No  -1 Not stated | -3/-1 = .  2 = 1 “No”  1 = 2 “Yes” |
| HS3 (Limitations in activities due to health problems) | Are you limited because of a health problem in activities people usually do?  Would you say you are … | 1 Severely limited  2 Limited but not severely  3 Not limited at all  -1 Not stated | -1=.  3=1 “No”  2=2 “Limited–not severely”  1=3 “Severely limited” |
| **Potential confounders:** Self-reported indicators of poor mental health.  *All questions in this section begin with “Over the last 2 weeks, how often have you been bothered by any of the following problems?...”* | | | |
| MH1A | ... little interest or pleasure in doing things | 1 Not at all  2 Several days  3 More than half the days  4 Nearly every day  -1 Not stated  -3 Proxy | -3/-1 = .  1=0  2=1  3=2  4=3 |
| MH1B | … feeling down, depressed or hopeless | 1 Not at all  2 Several days  3 More than half the days  4 Nearly every day  -1 Not stated  -3 Proxy | -3/-1 = .  1=0  2=1  3=2  4=3 |
| MH1C | ... trouble falling or staying asleep, or sleeping too much | 1 Not at all  2 Several days  3 More than half the days  4 Nearly every day  -1 Not stated  -3 Proxy | -3/-1 = .  1=0  2=1  3=2  4=3 |
| MH1D | ... feeling tired or having little energy | 1 Not at all  2 Several days  3 More than half the days  4 Nearly every day  -1 Not stated  -3 Proxy | -3/-1 = .  1=0  2=1  3=2  4=3 |
| MH1E | ... poor appetite or overeating | 1 Not at all  2 Several days  3 More than half the days  4 Nearly every day  -1 Not stated  -3 Proxy | -3/-1 = .  1=0  2=1  3=2  4=3 |
| MH1F | ... feeling negative about yourself or that you are a failure or have let yourself or your family down | 1 Not at all  2 Several days  3 More than half the days  4 Nearly every day  -1 Not stated  -3 Proxy | -3/-1 = .  1=0  2=1  3=2  4=3 |
| MH1G | … trouble concentrating on things, such as reading the newspaper or watching television | 1 Not at all  2 Several days  3 More than half the days  4 Nearly every day  -1 Not stated  -3 Proxy | -3/-1 = .  1=0  2=1  3=2  4=3 |
| MH1H | … moving or speaking so slowly that other people could have noticed. Or the opposite- being so fidgety or restless that they have been moving around a lot more than usual | 1 Not at all  2 Several days  3 More than half the days  4 Nearly every day  -1 Not stated  -3 Proxy | -3/-1 = .  1=0  2=1  3=2  4=3 |
| Generated variable: (Indicators of poor mental health) |  |  | The mental health questions come from the Patient Health Questionnaire eight-item depression scale (PHQ-8) (Kroenke et al., 2009). Using this scale, the scores can be summed to produce a total score between 0 to 24 points.  0-4 = 1 *none* (depressive symptoms)  5-9 = 2 *mild*  10-14 =3 *moderate*  15-19=4 *moderately severe*  20-24=5 *severe* |
| **Potential confounders:** Presence of comorbidities  *All questions in this section begin with “During the past 12 months, have you had any of the following diseases or conditions?...”* | | | |
| CD1A | … asthma (allergic asthma included) | 1 Yes  2 No  -1 Not stated | -1 = .  2 = 0 (“No”)  1 = 1 (“Yes”) |
| CD1B | … chronic bronchitis, chronic obstructive pulmonary disease or emphysema | 1 Yes  2 No  -1 Not stated | -1 = .  2 = 0 (“No”)  1 = 1 (“Yes”) |
| CD1C | … a myocardial infarction (heart attack) or chronic consequence of myocardial infarction | 1 Yes  2 No  -1 Not stated | -1 = .  2 = 0 (“No”)  1 = 1 (“Yes”) |
| CD1D | … a coronary heart disease or angina pectoris | 1 Yes  2 No  -1 Not stated | -1 = .  2 = 0 (“No”)  1 = 1 (“Yes”) |
| CD1E | … high blood pressure | 1 Yes  2 No  -1 Not stated | -1 = .  2 = 0 (“No”)  1 = 1 (“Yes”) |
| CD1F | … a stroke (cerebral haemorrhage, cerebral thrombosis) or chronic consequences of stroke | 1 Yes  2 No  -1 Not stated | -1 = .  2 = 0 (“No”)  1 = 1 (“Yes”) |
| CD1G | … arthrosis (arthritis excluded) | 1 Yes  2 No  -1 Not stated | -1 = .  2 = 0 (“No”)  1 = 1 (“Yes”) |
| CD1H | … a low back disorder or other chronic back defect | 1 Yes  2 No  -1 Not stated | -1 = .  2 = 0 (“No”)  1 = 1 (“Yes”) |
| CD1I | … a neck disorder or other chronic neck defect | 1 Yes  2 No  -1 Not stated | -1 = .  2 = 0 (“No”)  1 = 1 (“Yes”) |
| CD1J | … diabetes | 1 Yes  2 No  -1 Not stated | -1 = .  2 = 0 (“No”)  1 = 1 (“Yes”) |
| CD1K | … an allergy, such as rhinitis, eye inflammation, dermatitis, food allergy or other (allergic asthma excluded) | 1 Yes  2 No  -1 Not stated | -1 = .  2 = 0 (“No”)  1 = 1 (“Yes”) |
| CD1L | … cirrhosis of the liver | 1 Yes  2 No  -1 Not stated | -1 = .  2 = 0 (“No”)  1 = 1 (“Yes”) |
| CD1M | … urinary incontinence, problems in controlling the bladder | 1 Yes  2 No  -1 Not stated | -1 = .  2 = 0 (“No”)  1 = 1 (“Yes”) |
| CD1N | … kidney problems | 1 Yes  2 No  -1 Not stated | -1 = .  2 = 0 (“No”)  1 = 1 (“Yes”) |
| CD1O | … depression | 1 Yes  2 No  -1 Not stated | -1 = .  2 = 0 (“No”)  1 = 1 (“Yes”) |
| CD1P | … high blood lipids | 1 Yes  2 No  -1 Not stated | -1 = .  2 = 0 (“No”)  1 = 1 (“Yes”) |
| Generated variable: Presence of comorbidities |  |  | 0=1 “None”  1=2 “1 condition”  2/max=3 “2+ conditions” |

Supplementary Table 2. Country-level screening programme guidelines and the country-level data included in the study

|  | Country-level screening programme guidelines | | | | | | Country-level data included in the study | | | | | |
| --- | --- | --- | --- | --- | --- | --- | --- | --- | --- | --- | --- | --- |
| Countries (n=18) | Country-level screening programmes’ target age ranges | | | Country-level screening programmes’ target intervals | | | Country-level screening programme availability^a^ | | | Social Progress Index | Household out-of-pocket payments | GPs per 100,000 inhabitants^b^ |
|  | colorectal screening | mammography | cervical smear^h^ | colorectal screening | mammography | cervical  smear | colorectal screening | mammography | cervical smear |  |  |  |
| AT (Austria) | 40-80 (FOBT); 50+ (TC)^c^ | 45-69^c^ | 18+^c^ | 1 (FIT); 6 (TC)^c^ | 2^c^ | 1^c^ | 1^c^ | 2^c^ | 0^c^ | 86.99 | 17.92 | 152.94 |
| DE (Germany) | 50-74^c^ | 50-69^c^ | 20+^c^ | 1 (50-54 FOBT); 2 (55+ FOBT); 10 (55+ TC)^c^ | 2^c^ | 1^c^ | 1^c^ | 2^c^ | 1^c^ | 87.63 | 13.32 | 99.95 |
| DK (Denmark) | 50-74^c^ | 50-69^c^ | 23-59 (HPV test: 60-65)^c^ | 2^c^ | 2^c^ | 3 (23-59); 5 (60-64)^c^ | 2^c^ | 2^c^ | 2^c^ | 90.36 | 13.79 | 79.82 |
| EE (Estonia) | 60-69^c^ | 50-64 ^c^ | 30-59^c^ | 2^c^ | 2^c^ | 5^c^ | 1^c^ | 2^c^ | 2^c^ | 84.1 | 23.92 | 83.05 |
| EL (Greece) | 50-70^c^ | 40+^c^ | Sexual onset+^c^ | 2 (FOBT); 5 (TC)^c^ | 2 (40-49); 1 (50+)^c^ | —^c^ | 0^c^ | 0^c^ | 0^c^ | 79.57 | 33.64 | 44.14 |
| FI (Finland) | 60-69^c^ | 50-69^c^ | 30-64 (cytology or HPV test)^Rc^ | 2^c^ | 2^c^ | 5^c^ | 1^c^ | 2^c^ | 2^c^ | 89.89 | 17.42 | 135.22 |
| HU (Hungary) | 50-70^c^ | 45-64^c^ | 25-65^c^ | 2^c^ | 2^c^ | 3^c^ | 1^c^ | 2^c^ | 1^c^ | 77.22 | 27.79 | 74.73 |
| IS (Iceland) | —^d^ | 40–69^f^ | 23–65^e^ | —^d^ | 2^f^ | 3^e^ | 0^d^ | 2^g^ | 2^g^ | 89.39 | 15.55 | 59.63 |
| LT (Lithuania) | 50-74^c^ | 50-69^c^ | 25-59^c^ | 2^c^ | 2^c^ | 3^c^ | 1^c^ | 1^c^ | 1^c^ | 81.14 | 32.27 | 103.18 |
| LU (Luxembourg) | 55-74^c^ | 50-69^c^ | 18+^c^ | 2^c^ | 2^c^ | 1^c^ | 1^c^ | 2^c^ | 0^c^ | 87.55 | 9.59 | 89.55 |
| LV (Latvia) | 50-74^c^ | 50-69^c^ | 25-69^c^ | 1^c^ | 2^c^ | 3^c^ | 0^c^ | 2^c^ | 2^c^ | 78.97 | 35.11 | 71.53 |
| MT (Malta) | 55-66^c^ | 50-69^c^ | 25-35 (Piloting)^c^ | 2^c^ | 3^c^ | 3^c^ | 1^c^ | 2^c^ | 1^c^ | 81.93 | 34.13 | 82.33 |
| NL (Netherlands) | 55-75^c^ | 50-75^c^ | 30-64^c^ | 2^c^ | 2^c^ | 5^c^ | 1^c^ | 2^c^ | 2^c^ | 88.27 | 10.42 | 173.58 |
| NO (Norway) | —^d^ | 50-69^f^ | 25-33 (HPV test: 34-69)^e^ | —^d^ | 2^f^ | 3 (25-33); 5 (34-69)^e^ | 0^d^ | 2^g^ | 2^g^ | 90.56 | 13.92 | 96.52 |
| PT (Portugal) | 50-70^c^ | 45-74^Rc^ | 20-59^Rc^ | 2^c^ | 2^c^ | 3^c^ | 1^c^ | 1^c^ | 1^c^ | 84.11 | 30.61 | 282.7 |
| SE (Sweden) | 60-69^c^ | 40-74^c^ | 23-60^c^ | 2^c^ | 1.5-2^c^ | 3 (23-50); 5 (51-60)^c^ | 1^c^ | 2^c^ | 2^c^ | 88.83 | 13.71 | 62.07 |
| SI (Slovenia) | 50-74^c^ | 50-69^c^ | 20-64^c^ | 2^c^ | 2^c^ | Yearly x 2; then 3 yearly^c^ | 2^c^ | 1^c^ | 2^c^ | 85.14 | 11.66 | 67.71 |
| SK (Slovakia) | —^c^ | —^c^ | 23-64^c^ | —^c^ | —^c^ | Yearly x 2; then 3 yearly^c^ | 0^c^ | 0^c^ | 1^c^ | 79.24 | 19.16 | 41.42 |

Abbreviations: FOBT Faecal Occult Blood Test, FIT Fecal Immunochemical Test, TC Total Colonoscopy, HPV Human papillomavirus. ^a^(2) population-based organised programme, (1) ‘partial’ programme (pilot/rollout ongoing/regional programmes), (0) no programme. ^b^General practitioners (GP)s per 100,000 inhabitants. ^c^Ponti et al., 2017 ^d^Cardoso et al., 2020 ^e^Partanen et al., 2019 ^f^Peintinger, 2019. ^g^Jolidon et al., 2021. ^h^The screening programme age ranges for cervical screening refer to cytology/cervical smear instead of HPV unless otherwise stated. ^R^Countries with screening target age ranges that vary by regions or municipalities.

Supplementary Table 3. Missingness of variables

| Question Code | Variable description | No. of missing values | % Missing out of total observations |
| --- | --- | --- | --- |
| ***Outcome variable: Cancer screening utilisation*** | | | |
| PA5 | Last time of a faecal occult blood test | 1641 | 1.5% |
| PA6 | Last time of a colonoscopy | 954 | 0.9% |
| PA8 | Last time of a cervical smear test | 574 | 0.5% |
| PA7 | Last time of a mammography (breast X-ray) | 381 | 0.4% |
| ***(Individual-level) exposure variable: social support*** | | | |
| SS3 | How easy is it to get practical help from neighbours in case of need | 2631 | 2.5% |
| SS2 | Degree of concern shown by other people in what the person is doing | 1940 | 1.8% |
| SS1 | Number of close people to count on in case of serious personal problems | 1432 | 1.3% |
| ***Potential confounders: Social structure*** | | | |
| MAINSTAT | Self-declared employment status | 936 | 0.9% |
| HATLEVEL | Educational attainment level (highest level of education completed) | 780 | 0.7% |
| HHTYPE | Type of household | 1574 | 1.5% |
| ***Potential confounders: personal/family resources income, urbanisation*** | | | |
| UN2A | Need and affordability of medical care | 1345 | 1.3% |
| DEG_URB | Degree of urbanisation | 625 | 0.6% |
| HHINCOME | Net monthly equivalised income of the household | 2898 | 2.7% |
| ***Potential confounders: perceived needs*** | | | |
| HS1 | Self-perceived general health | 656 | 0.6% |
| HS2 | Long-standing health problem | 465 | 0.4% |
| HS3 | Limitation in activities because of health problems | 978 | 0.9% |
| ***Potential confounders: self-reported indicators of poor mental health*** | | | |
| MH1A | Extent of having little interest or pleasure in doing things over the last 2 weeks | 1275 | 1.2% |
| MH1B | Extent of feeling down, depressed or hopeless over the last 2 weeks | 1230 | 1.2% |
| MH1C | Extent of having trouble falling or staying asleep, or sleeping too much over the last 2 weeks | 1073 | 1.0% |
| MH1D | Extent of feeling tired or having little energy over the last 2 weeks | 1104 | 1.0% |
| MH1E | Extent of having poor appetite or overeating over the last 2 weeks | 1180 | 1.1% |
| MH1F | Extent of subject feeling negative about themselves, or feeling that they are a failure or have let themselves or their family down, over the last 2 weeks | 1336 | 1.3% |
| MH1G | Extent of having trouble concentrating on things, such as reading the newspaper or watching television, over the last 2 weeks | 1069 | 1.0% |
| MH1H | Extent of moving or speaking so slowly that other people could have noticed or being so fidgety or restless that they have been moving around a lot more than usual, over the last 2 weeks | 1271 | 1.2% |
| ***Potential confounders: evaluated comorbidities*** | | | |
| CD1A | Having asthma in the past 12 months (allergic asthma included) | 1231 | 1.2% |
| CD1B | Having chronic bronchitis, chronic obstructive pulmonary disease or emphysema in the past 12 months | 1379 | 1.3% |
| CD1C | Having a myocardial infarction (heart attack) or chronic consequence of myocardial infarction in the past 12 months | 1345 | 1.3% |
| CD1D | Having a coronary heart disease or angina pectoris in the past 12 months | 1461 | 1.4% |
| CD1E | Having high blood pressure in the past 12 months | 1165 | 1.1% |
| CD1F | Having a stroke (cerebral haemorrhage, cerebral thrombosis) or chronic consequences of stroke in the past 12 months | 1358 | 1.3% |
| CD1G | Having arthrosis (arthritis excluded) in the past 12 months | 1453 | 1.4% |
| CD1H | Having a low back disorder or other chronic back defect in the past 12 months | 1091 | 1.0% |
| CD1I | Having a neck disorder or other chronic neck defect in the past 12 months | 1194 | 1.1% |
| CD1J | Having diabetes in the past 12 months | 1452 | 1.4% |
| CD1K | Having an allergy, such as rhinitis, eye inflammation, dermatitis, food allergy or other (allergic asthma excluded) in the past 12 months | 1271 | 1.2% |
| CD1L | Having cirrhosis of the liver in the past 12 months | 1417 | 1.3% |
| CD1M | Having urinary incontinence, problems in controlling the bladder in the past 12 months | 1296 | 1.2% |
| CD1N | Having kidney problems in the past 12 months | 1367 | 1.3% |
| CD1O | Having depression in the past 12 months | 1451 | 1.4% |
| CD1P | Having high blood lipids in the past 12 months | 7739 | 7.2% |

Supplementary Table 4. Prevalence of individuals being overdue for cancer screening within each country.

|  | Overdue for cancer screening | | | |
| --- | --- | --- | --- | --- |
|  | Colorectal screening  N (%) | | Mammography  N (%) | Cervical smear  N (%) |
| Overall | 27,781 (42.7) | 10,950 (31.0) | | 7,121 (19.4) |
| Countries |  |  | |  |
| AT (Austria) | 1,543 (24.5) | 883 (26.5) | | 472 (12.0) |
| DE (Germany) | 2,895 (24.6) | 2,221 (35.0) | | 828 (15.3) |
| DK (Denmark) | 684 (21.6) | 342 (20.0) | | 482 (28.5) |
| EE (Estonia) | 1,273 (64.0) | 680 (57.2) | | 387 (31.0) |
| EL (Greece) | 2,468 (71.6) | 766 (41.4) | | 236 (13.4) |
| FI (Finland) | 2,113 (68.0) | 209 (12.1) | | 118 (8.4) |
| HU (Hungary) | 1,638 (70.5) | 547 (42.3) | | 275 (20.9) |
| IS (Iceland) | 640 (42.4) | 240 (30.3) | | 148 (15.2) |
| LT (Lithuania) | 946 (47.4) | 602 (48.7) | | 310 (24.6) |
| LU (Luxembourg) | 496 (27.4) | 190 (21.4) | | 176 (12.6) |
| LV (Latvia) | 1,431 (65.5) | 696 (52.7) | | 270 (16.7) |
| MT (Malta) | 1,006 (54.6) | 426 (44.1) | | 278 (26.6) |
| NL (Netherlands) | 1,177 (34.3) | 334 (19.2) | | 857 (43.0) |
| NO (Norway) | 2,068 (66.7) | 371 (24.5) | | 411 (21.2) |
| PT (Portugal) | 2,662 (39.8) | 869 (23.3) | | 1,022 (29.2) |
| SE (Sweden) | 2,219 (60.8) | 80 (4.3) | | 120 (5.6) |
| SI (Slovenia) | 1,175 (28.7) | 706 (32.5) | | 414 (15.8) |
| SK (Slovakia) | 1,347 (50.5) | 788 (48.9) | | 317 (21.2) |
